# Pure interstitial dup(6)(q22.31q22.31) – a case report

**DOI:** 10.1186/s13052-015-0113-y

**Published:** 2015-01-31

**Authors:** Frenny Sheth, Sunil Trivedi, Joris Andrieux, Jean-Louis Blouin, Jayesh Sheth

**Affiliations:** FRIGE’s Institute of Human Genetics, FRIGE House, Jodhpur Gam Road, Satellite, Ahmedabad, 380015 India; Laboratory of Medical Genetics, Jeanne de Flandre Hospital, CHRU, 59000 Lille, France; Laboratory of Molecular Diagnostics, Genetic Medicine, University Hospitals of Geneva, CH-1211 Geneva, Switzerland

**Keywords:** Dysmorpism, Neurological impairment, Duplication, 6q22.31, *TRDN*, *NKAIN2*

## Abstract

‘Pure’ interstitial duplication of chr6q is rare. The varying size of duplication encompassing 6q22.31 is associated with the expressivity of dysmorphism and autism. Here, we report a unique case with facial dysmorphism, developmental delay, complex neurological impairment and spasticity unrelated to autism. Genetic analysis by aCGH exhibited a 627–971 kb dup(6)(q22.31q22.31) encompassing *TRDN* and *NKAIN2* genes. The presence of the duplication was confirmed by quantitative PCR in the proband and phenotypically normal parents. With the current techniques, we cannot exclude presence of a deleterious homozygous point mutation in the proband where each copy would have been inherited from both parents.

## Background

Around 3.6% of the duplications observed in the DNA are mainly clustered within pericentromeric and subtelomeric regions [1]. Genomic DNA with segmental duplications are likely to be 1–200 kb in size and carry a high probability of encompassing repetitive sequences and coding genes [2]. Segmental duplication is described for all human chromosomes with slightly greater number of cases with maternal inheritance [3]. Duplication of long arm of chromosome 6 (chr6q) is rare. Most cases represent co-existence of an unbalanced translocation with other chromosome(s) that lead to a terminal duplication of chr6q with a partial monosomy of other chromosome/s. However, ‘Pure interstitial duplication of chr6q’ encompassing the larger segment is reported only in a few cases that provide clearly defined phenotypes affiliated to it [4,5]. Cases involving 6q22.31 duplication with another segmental aneusomy had phenotypic manifestations that are more associated to the later and not 6q22.31 [6,7]. Current case report presents an unusual case that portrays facial dysmorphism, severe developmental delay, complex neurological impairment and spasticity with 627–971 kb interstitial dup(6)(q22.31q22.31) as a sole observable anomaly inherited from either of the parents.

## Case report

**Figure 1:**
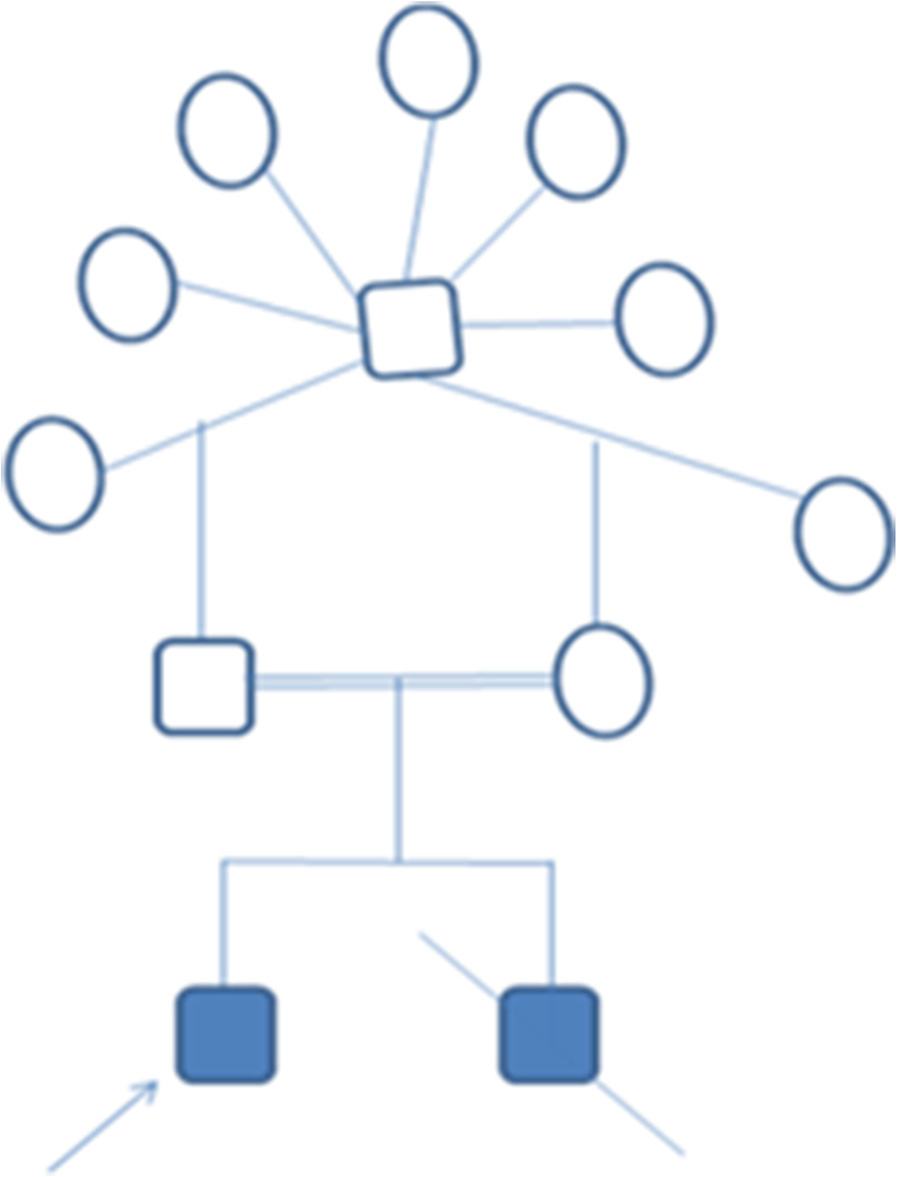
**Pedigree show parents as half sibs.**

**Figure 2:**
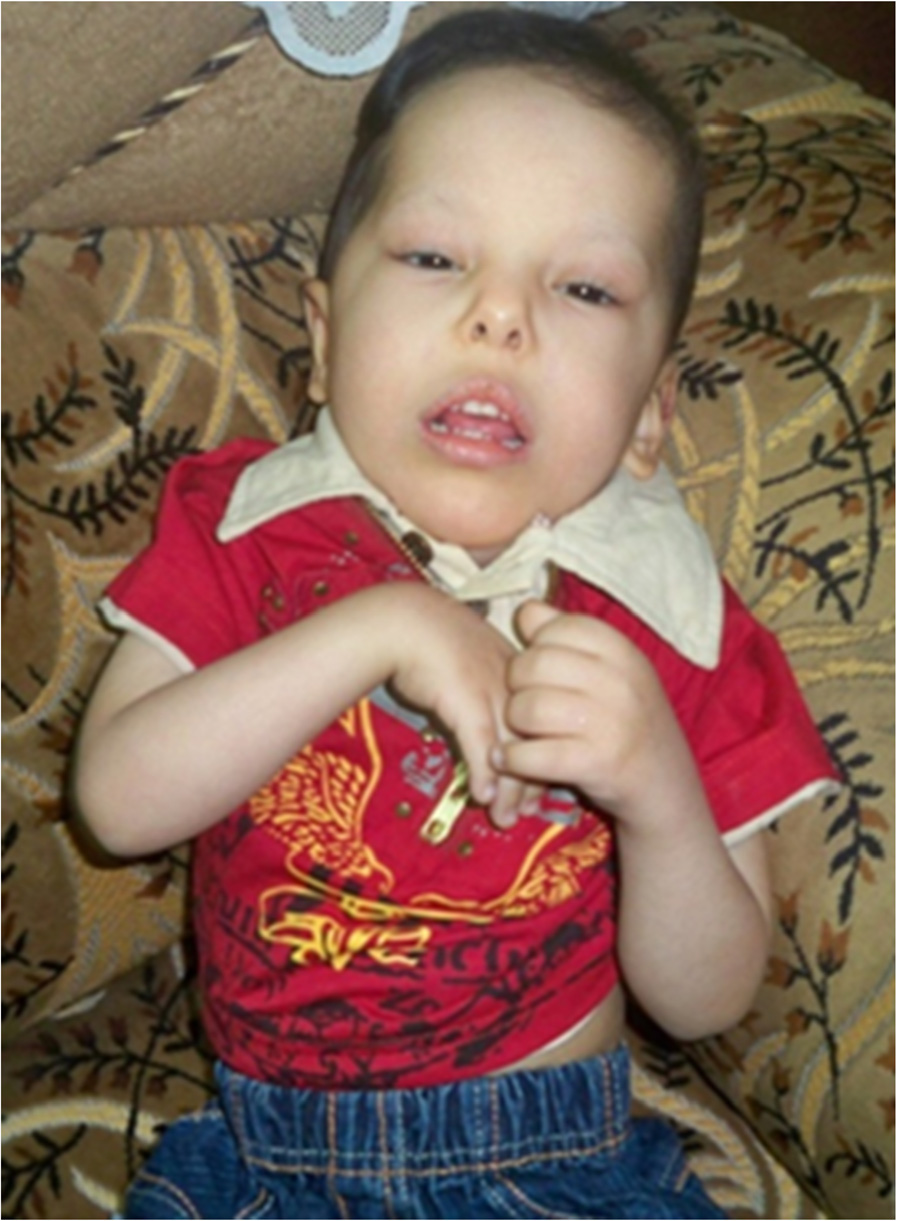
**Depiction of phenotype - proband.**

A 4½ years old boy was the first child born prematurely at 8 months by vaginal delivery to consanguineous parents who are half siblings (Figure 1). The age of the mother and father was 27 and 28 years respectively at the time of birth. Severe developmental delay and spacticity in the proband was first noticed at the age of 15 months. Physical examination revealed an asymmetrical face with dolicocephaly, large and prominent forehead and high anterior hairline. Eyes were small with arched eyebrows and scanty eyelashes. Hypertelorism, epicanthal folds and narrow palpabral fissures were also noted. Ears were low set, hypoplastic antitragus and lobule. Nose was short and stubby with wide nasal tip, broad nasal bridge, small anteverted nares, atresia choanae, hypoplastic alaenasi and thick columella. Philtrum was long and smooth. Upper lip was inverted V-shaped, down turned corners with wide and open mouth, thick lower lip, full cheeks, prominent mid face, underdeveloped nasolabial fold, mild retrognathis and broad jaw (Figure 2). He had short and stubby fingers with simian crease observed in the left palm. He was not able to sit, stand or walk without support at the age of presentation. Moreover, he could not recognize his parents. The younger sibling was also affected with the same clinical features and died at the age of 1 year.

**Figure 3:**
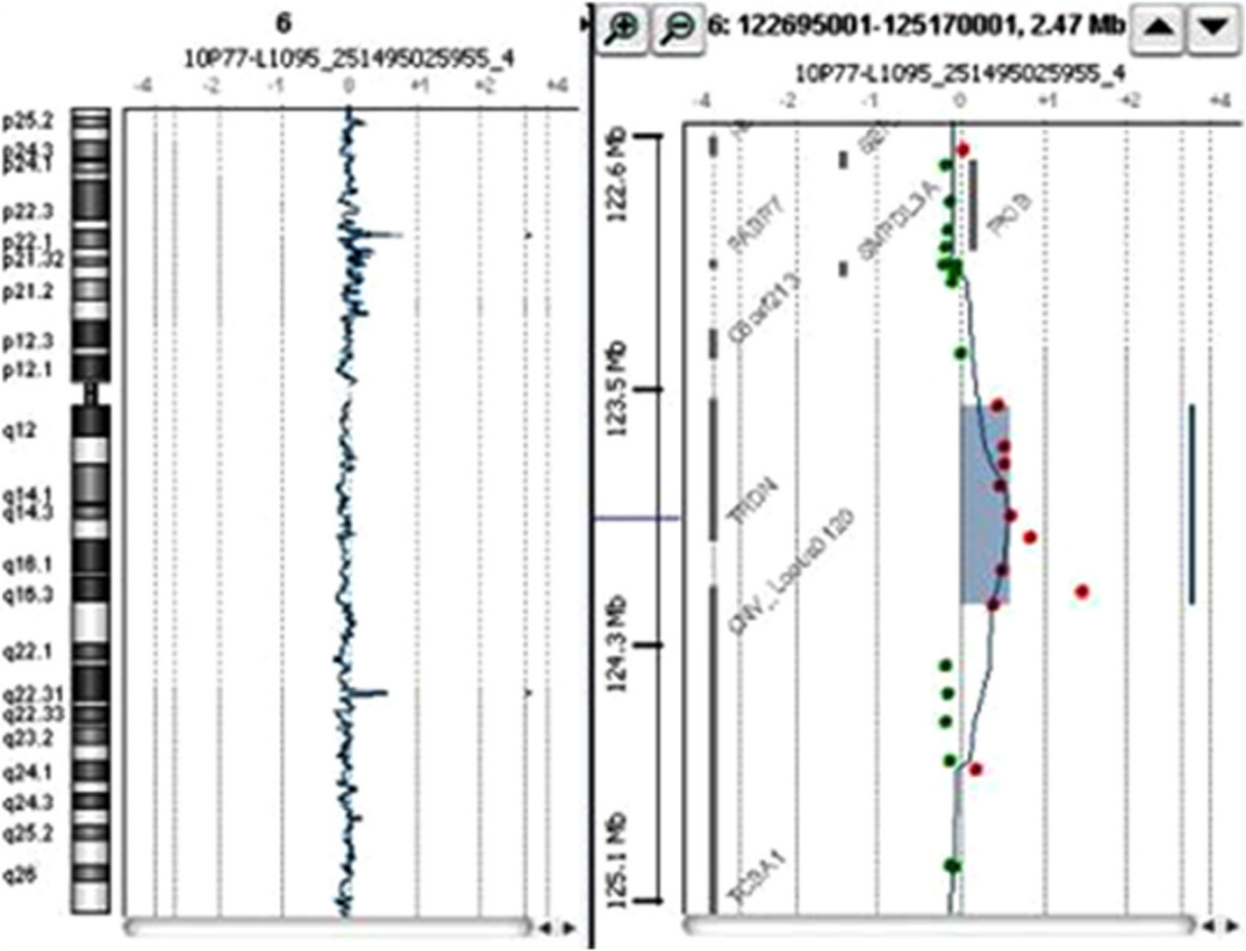
**Array-CGH showing 627-971 kb duplication on 6q22.31 region involving TRDN and NKAIN2 genes [(chr6:123,581,324-24,208,360)(hg18-build36)x3].**

**Figure 4:**
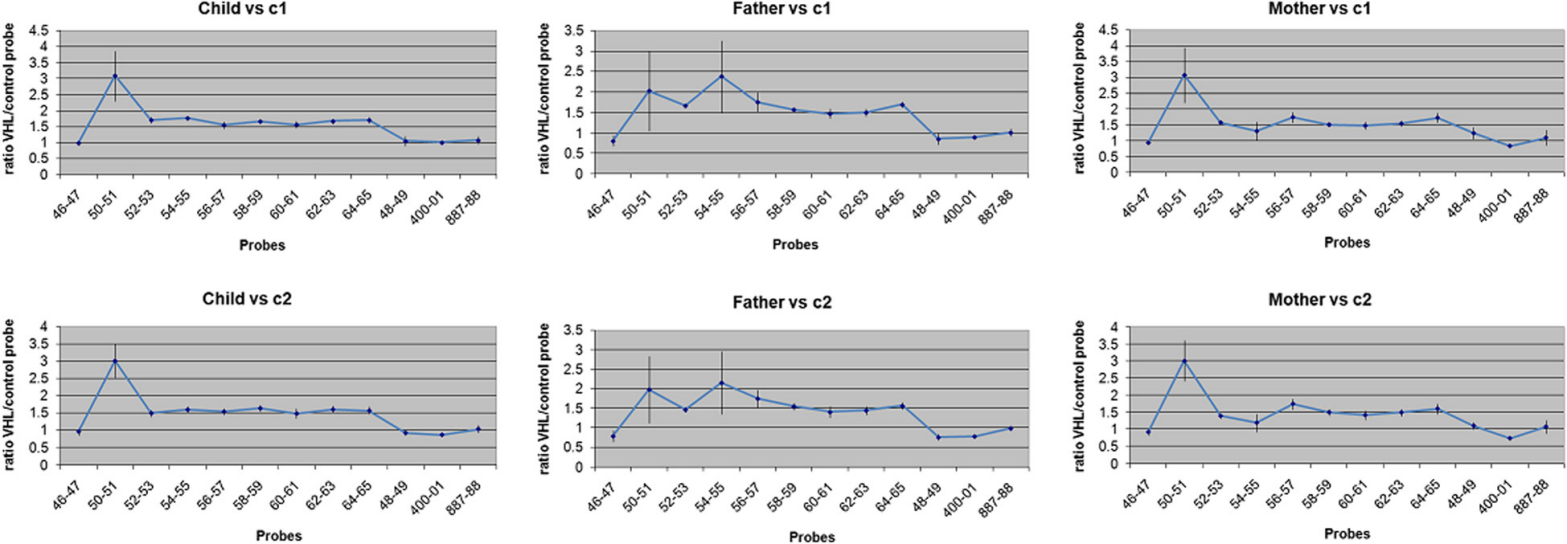
**Quantitative PCR results of the parents and proband.**

Conventional G-banding technique showed apparently normal chromosomal pattern [46,XY] at 550-band resolution. DNA from the proband and his parents was extracted from peripheral blood using the QIAamp DNA Blood Midi kit (Qiagen, Valencia, CA) to identify cryptic genomic imbalances. DNA concentration and quality was determined with NanoDrop (ND-1000 spectrophotometer and software; NanoDrop Technologies, Berlin, Germany). DNA copy number was detected with array-Comparative Genomic Hybridization (aCGH) following manufacturer’s recommendations using 60 K oligo probes approximately spaced at 40–100 kb intervals across the genome (Human Genome CGH microarray 60B kit, Agilent™). Sex matched genomic DNA (Promega Corporation, Madison, WI, USA) was used as a reference. Relative fluorescence intensity data was analyzed with the aCGH analysis software v3.4 (Agilent Technologies Inc., Santa Clara, CA, USA) by applying Z-score segmentation algorithm with a window size of 10 points to identify chromosome aberrations. We identified a 627–971 kb heterozygous duplication of 6q22.31 region in the proband [Figure 3]. Furthermore, the duplication was transmitted from either parent as both parents carried the same duplication as indentified using qPCR [Figure 4]. Quantity of the genomic DNA from the proband and parents was insufficient to carry out exome sequencing to identify point mutations that would have been missed by aCGH and test for their mode of inheritance. The minimal region affected by this duplication spans from chromosome 6 position 123,581,324 to 124,208,360 [(chr6: 123,581,324-124,208,360)(hg18 build36)x3]. This region does not overlap with any known CNVs in the Database of Genomic Variants [DGV] [8]. This region encompasses two genes, the entire coding region of *TRDN*, and the first exon of *NKAIN2*.

## Discussion

Micro-deletions and micro-duplications are relatively rare events, which arise during spermatogenesis or oogenesis. They might pass down disproportionately only for a few generations. They have been assigned to the ‘hotspot regions of the genome’ and are observed in several genetic disorders such as mental retardation (MR), developmental delay, schizophrenia, autism, neurocognitive disorders etc. [9,10]. Partial duplication of 6q with phenotypic alterations is reported only in few cases [5,11]. These reports showed that the duplication of 6q co-exist with other chromosomal abnormalities (chr16p and others) [6,7] where duplications were more common than deletions. Furthermore, the size of duplication is in direct correlation with the expression of clinical phenotypes. Scant reports of ‘Pure interstitial duplication of 6q’ are available in the literature [4,5]. The associated clinical features reported in most cases were intrauterine growth retardation (IUGR), hypertelorism, moderate facial dysmorphia (flat or depressed nasal bridge and anteverted nares), microcephaly, moderate psychomotor retardation, short fingers and cardiac anomaly linked to a varying degree of large sized duplication encompassing 6q22.31 [5]. The present case had a relatively smaller interstitial duplication (~0.62 Mb) and presented almost all of the above clinical features except cardiac anomaly. Furthermore, the proband had severe developmental and intellectual disability and spasticity. He was neither able to sit, speak, stand nor walk without external support at the age of the presentation. However, the younger sibling with all of the above phenotypic features died at the age of 1 year. No investigations were carried out in the younger sibling. Goh et al. [4] found similar features in dysmorphic siblings, which were trisomic for 6q22.1 to 6q23.3, representing a large duplication.

Sanders et al. [6] reported multiple recurrent *de novo* duplications that were strongly associated with autism. At least 8 patients were reported to harbour duplications in 6q22.31 region ranging from 0.03 to 0.62 Mb along with the other concurrent segmented aneusomy [7]. However, the patient presented in this report had a duplication of 0.62 Mb (627–971 kb; chr6:123,581,324‐124,208,360), only nearer to the previously reported regions. This region spans at least two genes (*TRDN* and *NKAIN2*), the entire coding region of *TRDN* [12] and the first exon of *NKAIN2. TRDN* (Triadin; OMIM No. 603283) with its alternatively spliced isoforms and differential expression is involved in excitation-contraction coupling of smooth and cardiac muscles as part of the calcium release complex in association with the ryanodine receptor. It has functions of (i) ion channel binding, (ii) protein binding and bridging, (iii) protein homo-dimerization activity and (iv) receptor binding functions. *NKAIN2* (Na+/K+ Transporting ATPase-interacting 2; OMIM No. 609758) is a trans-membrane protein that interacts with the beta subunit of a sodium/potassium-transporting ATPase. Truncation of *NKAIN2* has been described in patients with developmental delay [13] and complex neurological impairment [14]. The interstitial duplication detected in the present case could have been inherited from either of the parents.

Since both parents were phenotypically normal, it is highly likely that the proband has a homozygous deleterious point mutation, giving rise to the phenotypic expression of severe developmental, intellectual disability and spasticity. This hypothesis could be further supported by the observation of Froyen et al. in their study of 300 families with X-linked mental retardation (XLMR) identifying 6 overlapping duplications of about 320 kb involving four genes (*SMC1A, RIBC1, HSD17B10, HUWE1*) encompassing Xp11.2 in unrelated males [15]. In addition to the duplication, point mutation in *SMC1A* was shown to be associated with Cornelia de Lange syndrome with facial dysmorphism, mental retardation and growth deficit in childhood [16]. The syndromic form of mental retardation with choreoathetosis was shown to be associated with silent mutation in *HSD17B10* [17]*.* Moreover, point mutations of *HUWE1* gene leading to dose sensitisation may also partially be responsible for the phenotypes in cases with gene duplications, as shown by Froyen et al. [15].

Thus, the portrayed identical phenotype with severe morphological features presented here with relatively smaller pure interstitial dup(6)(q22.31q22.31) may additionally harbour deleterious point mutation, imparting a biologically pronounced effect which may be attributed to the high degree of consanguinity between parents.

## Consent

Written informed consent was obtained from the parents of the patient for publication of this Case Report and accompanying images.
